# Xpert Bladder Cancer Monitor for the Early Detection of Non-Muscle Invasive Bladder Cancer Recurrences: Could Cystoscopy Be Substituted?

**DOI:** 10.3390/cancers15143683

**Published:** 2023-07-19

**Authors:** Fernando Lozano, Carles X. Raventós, Albert Carrion, Carme Dinarés, Javier Hernández, Enrique Trilla, Juan Morote

**Affiliations:** 1 Department of Urology, Vall d’Hebron University Hospital, Universitat Autonoma Barcelona, 08035 Barcelona, Spain; carles.raventos@vallhebron.cat (C.X.R.); albert.carrion@vallhebron.cat (A.C.); enrique.trilla@vallhebron.cat (E.T.); juan.morote@vallhebron.cat (J.M.); 2 Pathology Department, Vall d’Hebron University Hospital, 08035 Barcelona, Spain; carme.dinares@vallhebron.cat (C.D.); javier.hernandez@vallhebron.cat (J.H.)

**Keywords:** bladder cancer, biomarker, surveillance

## Abstract

**Simple Summary:**

Non-muscle invasive bladder cancer (NMIBC) accounts for three quarters of newly detected bladder tumors. NMIBC can be treated conservatively with a bladder transurethral resection (bTUR), although recurrences are common despite adjuvant treatments. High-risk recurrent NMIBC can progress to muscle invasive bladder cancer (MIBC) and decrease survival. Therefore, close invasive surveillance, based on cystoscopy and washing cytology, is currently recommended, especially in high-risk recurrent tumors. Urine biomarkers have been investigated unsuccessfully to avoid or postpone the invasive surveillance of NMIBC. Xpert Bladder Cancer Monitor^®^ (XBM) is a new genetic urine biomarker that assesses the expression of five miRNA profiles. In the present study, XBM was not sensitive enough to detect all high-risk recurrences and avoid cystoscopy and washing cytology. However, false positive XBM results can predict early high-risk recurrences.

**Abstract:**

XBM was prospectively assessed in spontaneous urine collected just before flexible cystoscopy and washing cytology carried out within the first 2 years follow-up of 337 patients with NMIBC. Recurrences were pathologically confirmed in 49 patients (14.5%), 22 of them being high-risk (6.5%). The XBM sensitivity for detecting any type of recurrence was 69.4% and 63.6% in the cases of high-risk NMIBC. Negative predictive value (NPV) for XBM was 93% for all recurrences and 96.2% for high-risk recurrences. XBM could have avoided 213 invasive controls but missed the detection of 15 recurrences (30.6%)–8 of them of high-risk (36.4%). XBM false positive elevations were detected in 90 patients (26.7%), whereas 10 patients with the invasive method had a false positive result (3%), *p* <0.001. However, early detection of recurrences during the first year’s follow-up after an XBM false positive result was observed in 18 patients (20%). On the other hand, 19 recurrences were detected during this period among the rest of the patients (7.7%)—*p* = 0.003, and odds ratio (OR) 3.0 (95% CI 1.5–6.0). Regarding one-year follow-up recurrences, 10% were high-risk recurrences in the XBM false positive group and 3.2% in the rest of the patients—*p* = 0.021, and OR 3.3 (95% CI 1.2–8.9). Additionally, 11.3% of the patients without false positive results developed a recurrence, *p* = 0.897, for any recurrence, being 10% and 5.2%, respectively, and high-risk and low-risk recurrences, *p* = 0.506. After searching for the best XBM cutoff for detecting the 38 high-risk initial recurrences and the early high-risk recurrences after a one-year follow-up, a linear discriminant analysis (LDA) of 0.13 could have avoided 11.3% of cystoscopies and bladder wash cytologies, as this cutoff missed only 1 high-risk recurrence (2.6%). More extensive and well-designed studies will confirm if XBM can improve the surveillance of NMIBC.

## 1. Introduction

Bladder cancer is the sixth most common cancer in men, the seventeenth in women, and the tenth most frequent cancer in both sexes worldwide, with an estimated 573,278 new cases and 212,536 deaths in 2020 [1]. Europe has the highest incidence rate in the world, 11 cases per 100,000 persons per year, with the Spanish age-standardized incidence rate being one of the highest, 39 cases per 100,000 habitants in men [2,3]. In Spain, the crude mortality rate is 12/100,000, with significant differences in terms of gender comparison. In men, this mortality rate is 8.1 per 100,000, which is one of the highest in Europe [4,5].

Three quarters of newly diagnosed bladder tumors are non-muscle invasive bladder cancer (NMIBC), which are confined to the bladder mucosa (Ta stage and carcinoma in situ CIS) or the submucosa (T1 stage) [6]. NMIBC has a higher survival expectancy than muscle-invasive bladder cancer (MIBC) (T2-T4 stages), despite the treatment with radical cystectomy [7]. However, the overall recurrence rate of NMIBC is high, requiring frequent endoscopic controls with associated bothers and costs. Cystoscopy is an invasive procedure with a risk of side effects, such as painful micturition (50%), urinary frequency (37%), and macroscopic haematuria (19%) [8]. In addition, white light cystoscopy is not 100% sensitive to non-exophytic lesions or erythematous areas where CIS is suspected [9]. On the other hand, the cytological sensitivity is low, especially for low-grade tumors [10,11]. In addition, most biomarker studies use voided cytology rather than bladder washing, which is more sensitive and specific [12].

Cytology has a high interobserver variability and sometimes differentiates atypical changes, and inflammatory or infectious changes can be challenging for the pathologist [13]. The high NMIBC recurrence rate, usually defined by the European Organization for Research and Treatment of Cancer (EORTC) risk score, ranges from 31 to 78% [14]. It requires a precise surveillance program for early detection and treatment related to increased cancer-specific survival and overall survival [15]. The European Association of Urology (EAU) and the American Urological Association (AUA) guidelines recommend a combination of cystoscopy and cytology for the follow-up of patients with NMIBC [16,17], depending on its frequency on the EORTC risk group [14]. The Food and Drug Administration (FDA) approved urine biomarkers that have lower sensitivity and specificity [18], making their implementation in daily clinical practice challenging.

Research in new genetic urine biomarkers is increasing exponentially. However, most published studies compare voided cytology with the biomarker, which is not the real clinical gold standard. Furthermore, 2022 EAU Guidelines do not recommend using biomarkers in a surveillance protocol for high-risk NMIBC, because their performance cannot improve cystoscopy and cytology performance [19]. In the intermediate and low-risk groups, they suggest that, although there is no high-quality evidence, some of the newly available biomarkers could be used to replace or postpone cystoscopies. Recurrences in these groups are usually low grade, and biomarkers can identify with high sensitivity and negative predictive value the rare high-grade recurrence in this scenario [16].

Due to the lack of clinical alternatives to cystoscopy and cytology as surveillance methods for high-risk NMIBC, there is a trend towards developing new urinary biomarkers [20]. In fact, some of these available urine biomarkers have been recently approved by the FDA, but unfortunately, none have been incorporated into the clinical practice guidelines [21,22]. New modern biomarkers’ sensitivity and negative predictive value for high-grade recurrences reach over 90%, but their specificity and positive predictive value are usually low [23,24,25,26,27].

In recent years, due to their reproducibility, reliability, effortless performance, and objectivity, genetic biomarkers have become a promising investigation field in NMIBC surveillance. Genetic material quantification (DNA, RNA, miRNA, and lncRNA) and epigenetic changes, such as DNA hyper- and hypomethylation and histone mutations, have been studied [28]. Genetic biomarkers in bladder cancer have constantly been evolving and encompassing other phases of the disease due to its multiple possibilities for surveillance, screening, diagnosis, follow-up, treatment response, and prognosis. In addition, urine is an easy, harmless, fast liquid biopsy that contains stable genetic material.

The Xpert Bladder Cancer Monitor (XBM) test is a novel urinary biomarker that measures a panel of five micro-RNA targets (ANXA10, CRH y IGF2, ABL1, and UPK18) by qRT-PCR [29]. Micro RNAs (miRNA) are short, simple chains of 22 non-coding nu-cleotides that can induce posttranscriptional gene silencing by tethering an RNA-induced silencing complex to partly complementary sequence motifs in target mRNAs predominantly found within the 3′ untranslated regions [30]. MiRNAs are involved in multiple physiological and pathological events, including cell proliferation, survival, differentiation, growth, apoptosis, and immune activation [31]. miRNA expression in fluids like blood or urine is stable, allowing its quantification with qRT-PCR [32]. The first study exploring miRNA in bladder cancer was reported in 2007 by Gottardo et al. [33]; they identified the overexpression of ten miRNAs involved in the bladder carcinogenic pathway. Several pathological studies have suggested that low-grade and high-grade NMIBC have different molecular pathways activated, with low-grade tumors associated with the under-expression of some miRNAs. In contrast, the overexpression of miRNA is more common in high-grade tumors [34,35].

This study aimed to compare the urine genetic biomarker XBM with the gold standard methods of follow-up NMIBC based on white light flexible cystoscopy and urine cytology.

## 2. Materials and Methods

### 2.1. Design, Setting, and Participants

A prospective head-to-head comparison was made between XBM and the gold standard method of surveillance of NMIBC based on cystoscopy and washing bladder cytology [36] in 352 patients diagnosed between August 2018 and October 2020 in one academic institution. Follow-up evaluations were carried out for one year after XBM measurement to assess the early detection recurrences [37]. This project was approved by the institutional ethical committee (PRAG: 304/2018), and written consent was obtained from all participants.

### 2.2. Diagnostic Procedure

Bladder transurethral resection (bTUR) of the initial or recurrent tumor was performed. Randomized cold cup biopsies of the bladder and prostatic urethra were performed after bTUR in all initial bladder tumors as part of our hospital’s protocol, and in those patients with suspected high-risk tumors to assess simultaneous CIS [19]. Specimens were sent under the protocol to the pathology department. An experienced uro-pathologist analyzed them according to the 2017 T classification of urinary bladder cancer and graded them according to the 1973 and 2004/2016 World Health Organization grade classification [38].

### 2.3. Adjuvant Preventive Treatment for Recurrences

Postoperative intravesical Mitomycin C (40 mg) was instilled in the recovery room within the first 60 min after surgery if there were clinical indications and no postoperative contraindications based on the guideline’s recommendations [16]. Once NMIBC was diagnosed, intravesical recurrence prevention was scheduled according to the EORTC risk of recurrence and progression [39]. Nine patients received systemic immunotherapy in the context of a clinical trial (Table 1).

### 2.4. Follow-Up for Detection of Recurrences

The follow-up protocol included white light flexible cystoscopy under local anaesthesia in combination with bladder wash cytology obtaining 20 ccs of urine at the end of the procedure from the bladder neck with adequate fixation [16]. The frequency of follow-up cystoscopies and upper urinary tract imaging was based on the current EAU guidelines [16], with at least four cystoscopies and washing cytology per year in the first two years after bTUR in high-risk patients. In low-risk patients, cystoscopy and washing cytology were performed at three and nine months after bTUR and then yearly. In the intermediate-risk group, cystoscopy and washing cytology were performed every four months within the first two years.

Cytology was evaluated by dedicated cytopathologists. Falcon tubes were centrifuged for 5 min at 2800 rpm. The resulting cell pellets were resuspended in ThinPrep vials (Hologic Inc., Santa Clara, CA, USA) containing a methanol-based PreservCyt solution (Hologic Inc.) and processed using the ThinPrep 5000 System (Hologic Inc.). Cytological specimens were stained in Papanicolaou staining (QCA Química Clinica Aplicada S.A., Amposta, Spain) according to the Papanicolaou staining procedure. Upper urinary tract imaging with a CT scan urography was performed yearly in high-risk tumors [16].

### 2.5. Recurrence Suspicion and Diagnosis

The recurrence suspect was based either on bladder lesions detected by flexible cystoscopy and/or positive bladder wash cytology. The diagnosis was confirmed after the pathological analysis of bTUR material and/or bladder biopsies. Disease-negative patients had negative cystoscopy and washing cytology or negative pathological biopsy in those with suspicious lesions detected with the cystoscopy in the bladder.

In patients with positive bladder wash cytology but no visible tumor in the cystoscopy, investigation of extravesical locations using CT urography was performed. If no upper urinary tract tumor was detected by imaging, mapping biopsies of the bladder and prostatic urethra biopsy were performed based on the EAU Guidelines algorithm [40].

Recurrences were classified using the 2006 EORTC scoring model and divided into low, intermediate, and high risk [14].

### 2.6. XBM Assessment

The XBM biomarker was assessed prior to cystoscopy in spontaneously voided urine. Urine was collected the same day of the scheduled cystoscopy. Patients were requested to avoid first void in the morning and asked to collect at least 10 to 20 mL of their spontaneous micturition, preferably of the middle of the voiding. The Xpert Bladder Cancer Monitor^®^ (CE-IVD) was measured with the in vitro diagnostic Cepheid device (Sunnyvale Inc., Santa Clara, CA, USA). A 4.5 mL urine sample was added to the XBM urine transport reagent and mixed. Then, 4 mL of treated urine was transferred to the cartridge sample chamber, where cells in the urine sample were captured on a filter and lysed by sonication. The released nucleic acid was eluted and mixed with dry qRT-PCR reagents, and the solution was transferred to the reaction tube for RT-PCR and detection. The time to result was approximately 90 min. The XBM cartridges were preloaded with all reagents for the sample preparation, qRT-PCR analysis, and detection of five miRNA targets (ABL1, ANXA10, UPK1B, CRH, and IGF2). The cartridge also contained three controls: sample adequacy control (SAC), probe check control (PCC), and cepheid internal control (CIC) for sample-associated inhibition. The qualitative test provided a negative or positive result from the LDA algorithm, with a pre-set cutoff value at LDA ≥ 0.5 by the manufacturer, which used the cycle threshold results of these 5 miRNA targets.

Invalid XBM results were not considered, and missing data were not replaced. The XBM result was blinded for the urologist who performed the surveillance control and for the uropathologists.

### 2.7. XBM “False Positives” Follow-Up

All cohort participants were followed up for one year after the XBM assessment to evaluate early recurrences during this period and the possible anticipatory effect of the biomarker. All early recurrences and early high-risk recurrences were analyzed according to the XBM false positive, and incidents were compared to the rest of the cohort.

### 2.8. Statistical Analyses

Quantitative variables were expressed as the median and interquartile range (25 to 75 percentile). Qualitative variables were expressed as percentages. The association between quantitative variables was assessed with the Man Witney U test and between qualitative variables with the Chi-square test. The performance was analyzed with sensitivity, specificity, positive predictive value (PPV), negative predictive value (NPV), and accuracy. Avoided diagnostic procedures, missed recurrences, and high-risk recurrences were also analyzed. Binary logistic regression analysis assessed the predictive value of XBM, cystoscopy, and washing cytology for recurrences and high-risk recurrence. Odd ratios (OR) and 95% confidence intervals (95% CI) were also estimated. Finally, a *p*-value of less than 0.05 (two-tailed) was considered significant. This analysis was carried out with the SPSS v.25 (IBM, Armonk, NY, USA).

## 3. Results

### 3.1. Characteristics of Analyzed Population

**Figure 1:**
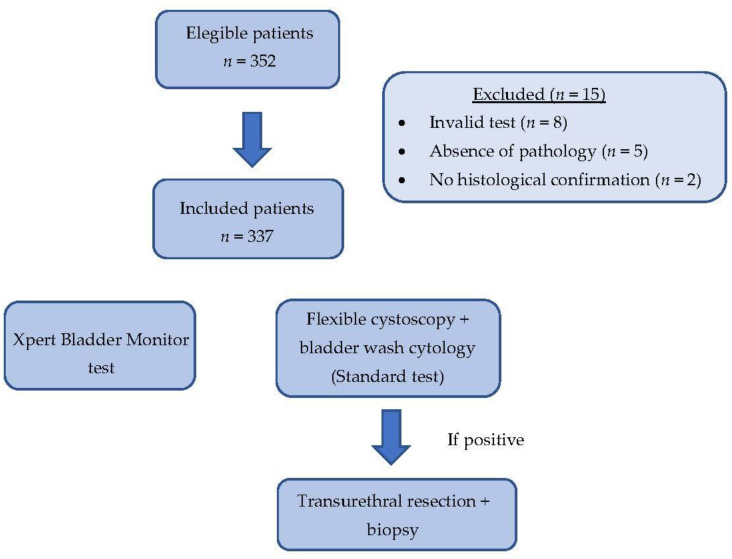
Flowchart of the patients’ recruitment process.

A total of 352 urine samples from patients with previous NMIBC were prospectively collected within the first 24 months of follow-up after their last NMIBC diagnostic performed by bTUR. Of these, eight patients were excluded due to invalid tests and five patients due to the absence of pathology. Another two patients were excluded due to the absence of histological confirmation of recurrence. Finally, 337 patients were included in the statistical analysis. The demographical and clinical characteristics of the patients are described in Table 1. A flowchart showing patient selection is described in Figure 1. 

The median age of the cohort was 73 years (interquartile range 65–80 years); 81.1% were males; 77.2% were smokers or former smokers; 68.3% were primary tumors, and 54.7% were high-grade tumors; 38% of the patients received postoperative mitomycin C; and 47% of the cohort received a full dose of BCG adjuvant therapy for one year.

During follow-up, 49 recurrences were detected (14.5% of the samples), with 5.5% being high-risk recurrences. During the one year follow-up, 33 recurrences were diagnosed—16 of them of high-risk. The median follow-up of the cohort was 13.5 months.

### 3.2. Performance of XBM

Sensitivity for any type of recurrences for XBM and cystoscopy plus bladder-wash cytology were 69.4% and 100%, respectively. Specificity for any type of recurrences of XBM and cystoscopy plus bladder-wash cytology were 68.8% and 96.5%, respectively. The NPV of XBM was 93%. Furthermore, the accuracy of XBM and cystoscopy plus bladder-wash cytology were 68.8% and 97%, respectively. In high-risk recurrences, the sensitivity of XBM and cystoscopy plus bladder-wash cytology were 63.6% and 100%, respectively. Specificity in the high-risk scenario for XBM and cystoscopy plus bladder-wash cytology were 65.1% and 88.3%, respectively. In high-risk, the NPV of XBM reached 96.2%. The accuracy of XBM and cystoscopy plus bladder-wash cytology for high-risk recurrences was 65% and 89%, respectively. These results are given in Table 2.

### 3.3. Prediction of Risk of Recurrence

Univariate XBM analysis, bladder wash cytology and flexible cystoscopy showed statistical significance for detecting recurrences for the three suspicion methods. A logistic multivariant regression was performed with the same three methods. Cystoscopy showed statistical significance for all high-risk recurrences and washing cytology only for high-risk recurrences (Table 3).

Univariate and multivariable analysis have been performed, selecting the three follow-up tests (Table 4). 

### 3.4. Follow Up on False Positives of XBM

False positive patients (positive biomarker and negative cystoscopy plus washing cytology, as defined from protocol) were followed up for one year to evaluate the possibility of an anticipatory effect of the biomarker in detecting early recurrences. Of the 90 (23.7%) false positive patients, 18 (20%) developed a recurrence that year, including 8 low-grade, 8 high-grade, and 2 upper urinary tract tumors. Statistically significant differences (*p* < 0.001) were found with XBM-negative patients that presented a recurrence rate of 6.1%. The odds ratio of patients with positive biomarkers but negative cystoscopy and cytology was 3 (1.494–6.023) and 3.3 (1.239–8.890) for high-risk disease.

### 3.5. Searching for a Clinically Useful XBM Cutoff

**Figure 2:**
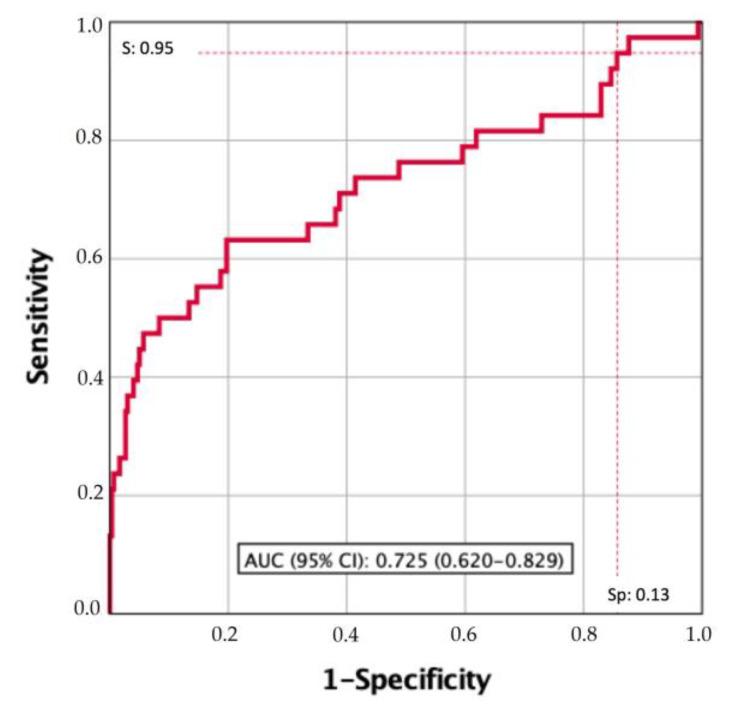
Discriminatory ability of XBM measurement to suspect high-risk recurrences at the time of its assessment and those diagnosed within the one-year follow-up. The 95% sensitivity of the XBM cutoff exhibits a 13% specificity, and the AUC is 0.725 (95% CI: 0.620–0.829).

The performance of XBM with the manufacturer LDA recommended a cutoff of 0.5 and exhibited a sensitivity of 63.6 for high-risk recurrences. Therefore, it was necessary to search for an XBM cutoff with higher sensitivity for high-risk recurrence discrimination at the time of its assessment and those early diagnosed high-risk recurrences within the first-year follow-up. The area under the curve (AUC) of XBM was 0.725 (95% CI: 0.620–0.829), Figure 2. Table 5 presents the LDA cutoff values of XBM of sensitivities between 100 and 75, its specificities, and the sensitivity and specificity corresponding to the 0.5 cutoff proposed by the manufacturer. We selected 0.1294 as the XBM cutoff with 95% sensitivity because it only missed 5% of high-risk recurrences, which is clinically reasonable for a biomarker that aspires to replace the gold standard surveillance protocol of NMIBC based on cystoscopy and cytology.

The sensitivity of XBM increased to 96.3%, but the specificity decreased to 13.7%. NPV was 92.1% and PPV 26.4%. For high-risk recurrences, the sensitivity was 97.4%, specifically 12.4%, NPV 97.4% and PPV 12.4%. The accuracy for XBM was 33.8% and 22% for all recurrences and high-risk recurrences, respectively. What seems more important from a clinical point of view is that 11.3% of cystoscopies and bladder wash cytologies could be avoided, as this cutoff missed only 2.6% of high-risk recurrences (Table 6).

The univariate analysis for predicting high-risk recurrences showed a significant XBM, cystoscopy, and bladder wash-cytology value. However, XBM was not an independent predictor in the multivariable analysis, as described on Table 7.

## 4. Discussion

Although increasing evidence suggests that new urine biomarkers have good performance for NMIBC surveillance, none has been consolidated as an actual alternative to the gold standard of cystoscopy and washing bladder cytology [41]. EAU Guidelines of 2021 [42] confirm that urinary markers cannot replace cystoscopy during follow-up or reduce the cystoscopy frequency. However, for the first time, the possibility of using biomarkers or bladder ultrasounds in patients initially diagnosed with TaG1-2/LG bladder cancer for surveillance has been noted in the case where cystoscopy was not possible or refused by the patient [43]. In the EAU Guidelines of 2022 [36], the potential role of four promising and commercially available urine biomarkers, Cx-Bladder [25], ADX-Bladder [44], Xpert Bladder [45], and EpiCheck [46], have been highlighted [3]. These markers have not been tested in randomized trials, so this novel approach cannot routinely replace cystoscopy during follow-up or lower cystoscopy frequency. Nevertheless, their high sensitivities and negative predictive values in the referenced studies, mainly for high-grade tumors and diseases, make these biomarkers attractive in avoiding cystoscopies in the follow-up of low/intermediate NMIBC [47]. This new step for biomarkers opens an alternative to the classical follow-up. It points out the option to individualize the surveillance protocols, considering the tumor characteristics and the patient’s age and performance status.

The first study of altered miRNA expression in bladder cancer was published in 2007 and detected the upregulation of 10 miRNAs [48] and miRNA as a urine biomarker for bladder cancer, as initially described by Weber et al. [49]. This genetic material expression in bladder cancer varies with intravesical treatment exposure and tumor grade. The profile of altered miRNAs differs between low- and high-grade tumors. In fact, high-grade NMIBCs share similar miRNA profiling to muscle-invasive tumors [32]. Since then, many miRNAs have been tested to detect and monitor bladder cancer patients. Although low-grade tumors usually have downregulation of many miRNAs, upregulation is more common in high-grade bladder cancer [31].

XBM was first validated in a multicentric study by Wallace et al. [50]. Since then, XBM has been tested in 10 studies [29,50,51,52,53,54,55] with more than 3000 patients. Overall sensitivity and specificity varied from 29.8 to 84% and 73.7 to 94.1%, respectively. The negative predictive value was between 83 and 96.5%, and the positive predictive value was between 44 and 90.9% [24]. One of the strengths of the XBM is that the test is automated. XBM can be assessed at the point of care and gives a fast result, with an easy and short hands-on sample preparation time of less than five minutes and single-use available disposable cartridges. It should, therefore, give the same result wherever patients are managed, whereas cytology results are pathologist-dependent [56]. All the previous studies made a direct comparison between both urine biomarkers, cytology, and XBM. However, none attempt to compare the whole follow-up protocol based on cytology plus cystoscopy was carried out [57].

This study was the first to compare XBM in a real clinical setting. The performance of XBM was compared to the gold standard follow-up of NMIBC, which included cystoscopy and bladder washing cytology. Considering this head-to-head comparison, XBM had a 68.8% sensitivity, 93% negative predictive value for all recurrences, and 96.2% for high-risk recurrences. This was in line with previous studies [51,52,53]. Nevertheless, compared to our daily clinical practice, XBM seemed unable to substitute the combination of cystoscopy and washing cytology by itself. Due to this and considering the biomarker’s ROC curve, we had carried an ad hoc analysis to find a better cutoff for the biomarker that may help detect all the high-risk recurrences during the follow-up. The counterpart of modifying the XBM pre-set threshold was decreasing the test’s specificity, which is associated with more negative cystoscopies, increasing the cost of the follow-up program and the patient’s anxiety due to a higher risk of false positive results. With the LDA threshold of 0.1294, the sensitivity of XBM increased to 96.3%, with a negative predictive value of 92.1%. Using this new threshold, 11.3% of the cystoscopies could be avoided with only a 2.6% chance of missing a high-risk recurrence, which are parameters comparable to the gold standard follow-up. On the other hand, specificity for all recurrences decreased to 13.7%

Biomarkers’ usefulness for the follow-up of NMIBC is based on four main criteria according to the ICUD-EAU International Consultation consensus [58]. First, they must be better—i.e., superior in clinical aspects to the standard tests (more sensitive, better NPV). Secondly, they must be simple, reproducible, and avoid complex infrastructures that complicate their standardization and dissemination. Thirdly, they should be faster or, at least, the biomarkers’ results should be available in a short period time. Lastly, they must be cheaper or economically similar to the gold standard combination. Cost-efficacy studies of biomarkers are complex and usually based on non-clinical models [59]. Due to the significant variability between countries’ health care systems and the complexity of the evaluation of indirect costs of the cystoscopy (urologist time, nurse, material, and theatre time), the comparison between the standard protocol and biomarker follow-up had a high risk of bias. Nam et al. [60] demonstrated that a follow-up based exclusively on biomarkers was economically more efficient than the standard follow-up. However, their study did not take into account the costs and profiles of the most recent biomarkers. After evaluating the biomarker in a cohort study, a randomized control trial should be carried out comparing the gold standard method, cystoscopy plus cytology, and the urine biomarker in a real clinical scenario. If results confirm the non-inferiority performance, the next step to establish a new protocol based on biomarkers follow-up, subsidized by the National Health Care System, should include a cost-efficiency study.

Besides the four main criteria suggested by the ICUD-EAU International Consultation, another characteristic should be considered when changing the paradigm of NMIBC surveillance. Most of the biomarkers’ studies presuppose that patients will agree to change their follow-up protocol because this new tool is essentially as sensitive as cystoscopy and cytology. Flexible cystoscopy continues to be an invasive procedure, costly, bothersome, and painful for the patients; it increases the risk of urinary tract infection, and over 60% of the patients experience adverse psychological effects related to the procedure [61,62]. Moreover, this method has limitations for detecting small and flat lesions (post-TURBT, CIS) [63]. Nevertheless, a study by van Osch et al. [64] confirmed that half of the patients would not replace cystoscopy unless the biomarker was 100% sensitive, and 85% of the patients would only change if the biomarker performance achieved 99% of sensitivity. Moreover, research by Shen Tan et al. [65] confirmed that although patients experienced bothersome symptoms after cystoscopy, with hematuria in 51% or dysuria in 69% of them, they are more confident with a visual diagnosis of the bladder and would only accept the change if the biomarker had at least the same sensitivity as cystoscopy. This situation may not reflect a complete understanding of the concept of patient sensitivity and their fear of the possibility of missing a recurrence. An actual clinical scenario should be transmitted to the patients and the differences in the profile of the biomarkers; in the low-risk group, missing a single, small bladder tumor does not impact the patient´s overall survival or cancer-specific survival. However, in the high-risk group, early detection is mandatory, and a biomarker will never substitute the gold standard if it cannot detect small high-grade recurrences.

A positive biomarker result had been demonstrated to increase the cystoscopies’ detection rate [32]. When analyzing the longitudinal effect, definitions are contradictory. Some recent studies suggested that enhanced image cystoscopies may improve the detection of small or plain lesions, hence decreasing false positive biomarker results [66]. It has also been suggested that the Studer’s algorithm should be applied to exclude extravesical recurrences in cases with negative cystoscopy but positive biomarkers, including cytology [67]. A previous XBM study by Cowan et al. [68] has explored this possible anticipatory effect. In their research, 131 patients were followed up for 1 year with negative cystoscopy, independently of the cytology result, comparing those with positive and negative biomarkers. It was found that the former had an increased risk of developing a high-grade recurrence.

As a secondary objective of this study, our patients were followed up for one year to evaluate if a false positive biomarker could have any anticipatory information about the risk of recurrence. Some positive urine biomarkers are associated with an increased risk of recurrence and progression, even if the patient had a negative cystoscopy at the time of the determination [69]. Gopalakrishna et al. [70] tried to define the positive anticipatory result for bladder cancer. They assumed a period of one year to define the possible anticipatory result. They demonstrated that a positive urine test does not always mean future tumor recurrence. Only 75% of the positive cytologies or 40% of the positive UroVysion FISH tests developed a tumor within one year.

It was unclear in the literature if this anticipatory effect should make us change our clinical practice protocol with these patients, such as a more intense cystoscopy follow-up, random biopsies, and upper urinary tract endoscopic exploration. In our cohort, false positive XBMs were followed for one year. The recurrence rate in the false positive XBM group was 20%, while only 6.1% of the patients with an XBM negative test experienced a recurrence. That means that when the biomarker was positive but gold standard methods were negative, the patient had a statistically increased odds ratio of 3 (1.5–6) with *p* < 0.003 for all recurrences, and an odds ratio of 3.3 (1.2–8.9) with *p* < 0.02 for high-risk recurrences within the following year. This study opened an option for a new interpretation of the genetic urine biomarkers. Until now, a negative or positive result was read transversally. Nevertheless, the biomarkers’ field still has many open questions, and one of those is how a positive result without macroscopic translation must be read. The one year follow-up in our study demonstrated that patients with positive XBM had a higher risk of developing a recurrence, and this information should be taken into account by clinicians to adjust the follow-up scheme. However, this data should be interpreted carefully, and information given to the patient must be based on evidence-based follow-up protocols to avoid anxiety and changes in the follow-up protocol weighted due to the lack of clear perspective and high-level data. If that information should change our protocol is a question that cannot be answered nowadays. Moreover, we assumed that the definition of a false positive biomarker’s result was based on a negative cystoscopy and cytology. Future studies may include a negative upper urinary tract study with a CT scan and/or bladder random biopsies with prostatic urethral biopsies as a confirmatory protocol.

This study had some limitations. On the one hand, negative and positive predictive values are parameters influenced by disease incidence prevalence. Selecting patients during the first two years of follow-up was not the actual clinical scenario and can increase the incidence prevalence of recurrences, hence overestimating both parameters. Moreover, the low recurrence rate in our study (14.5% of the cohort), may have had an impact on the difficulty of finding statistically significant differences. On the other hand, patients were monitored with cystoscopy, following the 2021 EAU Guidelines. The mean recurrence size was 0.8 cm (0.3–1.6 cm), which could be considered a low tumor burden detected by biomarkers. Different cytology specimens have also been used, such as spontaneous miction urine to avoid invasive follow-up methods for XBM and bladder wash cytology for cytopathologic study. That means that different samples were compared for urine biomarkers. Interestingly, although these are two different methods of obtaining the urine sample, sensitivity and NPV were still high for XBM.

Neither our study nor the previous papers had a real clinical design randomizing cystoscopy and XBM, which can complicate the implementation of daily clinical practice. Alternating biomarkers and cystoscopy plus cytology is another approach our study had not explored.

## 5. Conclusions

XBM had demonstrated an acceptable sensitivity and negative predictive value for high-risk recurrences of NMIBC. A change in the threshold proposed by the manufacturer increased its sensitivity and negative predictive value in our series, with a slight decrease of specificity. Although XBM did not guarantee the 100% prediction of high-risk recurrences, its positive result in absence of cystoscopic or cytologic confirmation of bladder tumors increased the probability of developing a tumor recurrence in the next year of its determination. Longitudinal and randomized studies are needed to identify the exact role of XBM in the surveillance of NIMBC.

## Figures and Tables

**Table 1:** Demographic and clinical characteristics of the study cohort.

Parameter	Value
**Median age, years (IQR)**	73 (65–80)
**Gender, *n* (%)**	
Male	274 (81.1)
Female	64 (18.9)
**Smoke habit, *n* (%)**	
Smoker/former smoker	261 (77.2)
Non- smoker	77 (22.8)
**Type of tumour, *n* (%)**	
Primary	231 (68.3)
Recurrence within one year follow-up	64 (18.9)
Recurrence later than one year follow-up	43 (12.7)
**Previous number of recurrences**	
One	63 (58.9)
Two or more	44 (41.1)
EORTC * risk of recurrence and progression, *n* (%)	
Low	84 (24.9)
Intermediate	67 (19.8)
High	187 (55.3)
**Pathological stage, *n* (%)**	
Ta	156 (47.1)
T1	115 (34)
CIS **	12 (3.6)
Tx	55 (16.3)
**Pathological grade, *n* (%)**	
Low	153 (45.3)
High	185 (54.7)
**Adjuvant treatment, *n* (%)**	
Postoperative Mytomicin C	128 (38)
No	152 (45)
BCG ***	159 (47)
Mytomicin C	18 (5.3)
Systemic immunotherapy	9 (2.7)
**Recurrences diagnosed at the time of XBM assessment, *n* (%)**	
Any recurrence	49 (14.5)
High-risk recurrence	22 (6.5)
No recurrence	266 (79)
**Recurrences diagnosed within one year follow-up, *n* (%)**	
Any recurrence	33 (9.8)
High-risk recurrence	16 (5.6)

* EORTC = European Organisation for Research and Treatment of Cancer; ** CIS = carcinoma in situ; *** BCG = Bacille Calmette-Guerin.

**Table 2:** Incidence percentage of grade and stage diagnosed using the Xpert Bladder Monitor.

Variable	Xpert Bladder Monitor	
**Grade**	Positive	Negative
Low Grade, *n* (%)	20/27 (74.1)	7/27 (25.9)
High Grade, *n* (%)	14/22 (63.6)	8/22 (36.4)
**Pathological stage**		
Ta, *n* (%)	19/27 (70.4)	8/27 (29.6)
Tx, *n* (%)	6/7 (85.7)	1/7 (14.3)
Tis *, *n* (%)	4/4 (100)	0/4 (0)
T1, *n* (%)	3/9 (33.3)	6/9 (66.7)
T2, *n* (%)	2/2 (100)	0/2 (100)

* Tis: Cacinoma in situ.

**Table 3:** Performance of XBM compared with cystoscopy and washing cytology for the suspicion of any type of recurrence and high-risk recurrences at the time of XBM assessment.

Parameter	All Recurrences		High-Risk Recurrences	
	XBM	Cystoscopy and Washing Cytology	XBM	Cystoscopy and Washing Cytology
Sensitivity, *n* (%)	34/49 (69.4)	49/49 (100)	14/22 (63.6)	22/22 (100)
Specificity, *n* (%)	198/288 (68.8)	278/288 (96.5)	205/315 (65.1)	278/315 (88.3)
Positive predictive value, *n* (%)	34/124 (27.4)	49/59 (83.1)	14/124 (11.3)	22/59 (37.3)
Negative predictive value, *n* (%)	198/213 (93.0)	278/278 (100)	205/213 (96.2)	278/278 (100)
Accuracy, *n* (%)	232/337 (68.8)	327/337 (97.0)	219/337 (65.0)	300/337 (89)
Avoided diagnostic procedures, *n* (%)	213/337 (63.2)	0 (0)	213/337 (63.2)	0 (0)
Missed recurrences, *n* (%)	15/49 (30.6)	0 (0)	8/22 (36.4)	0 (0)

**Table 4:** Univariate and multivariable analysis of XBM, cystoscopy, and washing cytology as suspicion methods for predicting any type of recurrence and high-risk recurrences diagnosed at the time of XBM assessment.

Method of Suspicion	Univariate Analysis		Multivariable Analysis	
	Odd Ratio (95% CI)	*p* Value	Odd Ratio (95% CI)	*p* Value
**For any type of recurrence**				
XBM	4.987 (2.586–9.616)	=0.001	3.585 (0.820–15.675)	=0.090
Cystoscopy	615.524 (153.624–2466.212)	<0.001	1517.105 (175.210–13136.239)	<0.001
Washing cytology	15.975 (4.700–54.296)	<0.001	100.409 (7.207–1398.817)	=0.110
**For high-risk recurrences**				
XBM	3.261 (1.327–8.014)	=0.007	0.723 (0.226–2.312)	=0.585
Cystoscopy	52.343 (14.720–186.124)	=0.001	53.712 (13.243–217.851)	<0.001
Washing cytology	24.033 (7.186–80.377)	=0.001	22.473 (3.530–143.048)	=0.001

**Table 5:** Specificities corresponding to the cutoff sensitivities of 1.00 to 0.75 of XBM for the suspicion of high-risk recurrence at the time of its assessment and those diagnosed within the one-year follow-up. Sensitivity and specificity corresponding to the cutoff recommended by the manufacturer (LDA = 0.5) are 63% and 66.6%, respectively.

Sensitivity (%)	Specificity (%)	Cutoff
100	7.1	0.1117
95	13.4	0.1294
90	15.4	0.1459
85	17.1	0.6661
80	38.1	0.2950
75	51.2	0.3950
63	66.6	0.5000

**Table 6:** Performance of XBM (using the 0.1294 cutoff) compared with cystoscopy and washing cytology for the suspicion of any type of recurrence and high-risk recurrences at the time of XBM assessment and within one year follow-up.

Parameter	All Recurrences		High-Risk Recurrences	
	XBM	Cystoscopy and Washing Cytology	XBM	Cystoscopy and Washing Cytology
Sensitivity, *n* (%)	79/82 (96.3)	50/82 (61.0)	37/38 (97.4)	24/38 (63.2)
Specificity, *n* (%)	35/255 (13.7)	246/255 (96.5)	37/299 (12.4)	264/299 (88.3)
Positive predictive value, *n* (%)	79/299 (26.4)	50/59 (84.7)	37/299 (12.4)	24/59 (40.7)
Negative predictive value, *n* (%)	35/38 (92.1)	246/278 (88.5)	37/38 (97.4)	264/278 (95.0)
Accuracy, *n* (%)	114/337 (33.8)	296/337 (87.8)	74/337 (22.0)	288/337 (85.5)
Avoided diagnostic procedures, *n* (%)	38/337 (11.3)	278/337 (82.5)	38/337 (11.3)	278/337 (82.5)
Missed recurrences, *n* (%)	3/82 (3.7)	32/482 (39.0)	1/38 (2.6)	14/38 (36.8)

**Table 7:** Univariate and multivariable analysis of XBM, cystoscopy, and washing cytology as suspicion methods to predict any type of recurrence, and high-risk recurrences diagnosed at the time of XBM assessment and those diagnosed within one year follow-up.

Method of Suspicion	Univariate Analysis		Multivariable Analysis	
	Odd Ratio (95% CI)	*p* Value	Odd Ratio (95% CI)	*p* Value
**For any type of recurrence**				
XBM	4.189 (1.253–14.004)	=0.090	2.178 (0.567–8.369)	=0.257
Cystoscopy	55.729 (22.200–139.897)	<0.001	49.818 (19.623–126.477)	<0.001
Washing cytology	11.667 (3.127–43.522)	<0.001	7.762 (1.597–37.711)	=0.110
**For high-risk recurrences**				
XBM	5.225 (0.96–39.227)	=0.052	2.644 (0.337–20.714)	=0.355
Cystoscopy	10.307 (4.932–21.540)	< 0.001	8.182 (3.766–17.773)	<0.001
Washing cytology	15.680 (4.824–59.968)	< 0.001	9.504 (2.515–35.919)	=0.001

## Data Availability

The data presented in this study is available on request from the corresponding author.

## References

[1] Sung H., Ferlay J., Siegel R.L., Laversanne M., Soerjomataram I., Jemal A., Bray F. (2021). Global Cancer Statistics 2020: GLOBOCAN Estimates of Incidence and Mortality Worldwide for 36 Cancers in 185 Countries. CA Cancer J. Clin..

[2] Bray F., Ferlay J., Soerjomataram I., Siegel R.L., Torre L.A., Jemal A. (2018). Global cancer statistics 2018: GLOBOCAN estimates of incidence and mortality worldwide for 36 cancers in 185 countries. CA Cancer J. Clin..

[3] Ferlay J., Colombet M., Soerjomataram I., Mathers C., Parkin D.M., Piñeros M., Znaor A., Bray F. (2019). Estimating the global cancer incidence and mortality in 2018: GLOBOCAN sources and methods. Int. J. Cancer.

[4] Bernal-Pérez M., Souza D., Romero-Fernández F., Gómez-Bernal G., Gómez-Bernal F. (2013). Estimación de las proyecciones del cáncer de vejiga en España. Actas Urol Esp.

[5] Antoni S., Ferlay J., Soerjomataram I., Znaor A., Jemal A., Bray F. (2017). Bladder Cancer Incidence and Mortality: A Global Overview and Recent Trends. Eur. Urol..

[6] Compérat E., Larré S., Rouprêt M., Neuzillet Y., Pignot G., Quintens H., Houede N., Roy C., Durand X., Varinot J. (2015). Clinicopathological characteristics of urothelial bladder cancer in patients less than 40 years old. Virchows Arch..

[7] Chen J., Zhang H., Sun G., Zhang X., Zhao J., Liu J., Shen P., Shi M., Zeng H. (2018). Comparison of the prognosis of primary and progressive muscle-invasive bladder cancer after radical cystectomy: A systematic review and meta-analysis. Int. J. Surg..

[8] Burke D.M., Shackley D.C., O’Reilly P.H. (2002). The community-based morbidity of flexible cystoscopy. BJU Int..

[9] Herr H.W., Donat S.M., Dalbagni G. (2002). Correlation of Cystoscopy with Histology of Recurrent Papillary Tumors of the Bladder. J. Urol..

[10] Karakiewicz P.I., Benayoun S., Zippe C., Ludecke G., Boman H., Sanchez-Carbayo M., Casella R., Mian C., Friedrich M.G., Eissa S. (2006). Institutional variability in the accuracy of urinary cytology for predicting recurrence of transitional cell carcinoma of the bladder. BJU Int..

[11] Bensalah K., Montorsi F., Shariat S.F. (2007). Challenges of Cancer Biomarker Profiling {A Figure Is Presented}. Eur. Urol..

[12] Sarosdy M., deVere White R.D., Soloway M.S., Sheinfeld J., Hudson M., Schell-er P.F., Jarowenko M., Adams G., Blumenstein B.A., Ellis W.J. (1995). Results of A Multicenter Trial Using The Bta Test to Monitor for and Dmxvose Recurrent Bladder Cancer. J. Urol..

[13] Raitanen M.-P., Aine R., Rintala E., Kallio J., Rajala P., Juusela H., Tammela T.L., FinnBladder Group (2002). Differences Between Local and Review Urinary Cytology in Diagnosis of Bladder Cancer. An Interobserver Multicenter Analysis. Eur. Urol..

[14] Soukup V., Čapoun O., Cohen D., Hernández V., Burger M., Compérat E., Gontero P., Lam T., Mostafid A.H., Palou J. (2020). Risk Stratification Tools and Prognostic Models in Non–muscle-invasive Bladder Cancer: A Critical Assessment from the European Association of Urology Non-muscle-invasive Bladder Cancer Guidelines Panel. Eur. Urol. Focus.

[15] Hollenbeck B.K., Dunn R.L., Ye Z., Hollingsworth J.M., Skolarus T.A., Kim S.P., Montie J.E., Lee C.T., Wood D.P., Miller D.C. (2010). Delays in diagnosis and bladder cancer mortality. Cancer.

[16] Babjuk M., Burger M., Compérat E., Gontero P., Mostafid A.H., Palou J., Van Rhijn B.W.G., Rouprêt M., Shariat S.F., Sylvester R. (2022). Non-Muscle-Invasive Bladder Cancer (TaT1 and CIS) EAU Guidelines. Eur. Urol..

[17] Daneshmand S., Konety B.R. (2016). American Urological Association (AUA) Guideline American Urological Association Non-Muscle Invasive Bladder Cancer.

[18] Soria F., Droller M.J., Lotan Y., Gontero P., D’andrea D., Gust K.M., Rouprêt M., Babjuk M., Palou J., Shariat S.F. (2018). An up-to-date catalog of available urinary biomarkers for the surveillance of non-muscle invasive bladder cancer. World J. Urol..

[19] Compérat E., Gontero P., Liedberg F., Masson-Lecomte A., Mostafid A.H., Palou J., Van Rhijn B.W.G., Rouprêt M., Shariat S.F., Sylvester R. (2022). Non-Muscle-Invasive Bladder Cancer (TaT1 and CIS) EAU Guidelines On.

[20] Lotan Y., Black P.C., Caba L., Chang S.S., Cookson M.S., Daneshmand S., Kamat A.M., McKiernan J.M., Pruthi R.S., Ritch C.R. (2018). Optimal Trial Design for Studying Urinary Markers in Bladder Cancer: A Collaborative Review. Eur. Urol. Oncol..

[21] Van Rhijn B.W., van der Poel H.G., van der Kwast T.H. (2005). Urine Markers for Bladder Cancer Surveillance: A Systematic Review. Eur. Urol..

[22] Mbeutcha A., Lucca I., Mathieu R., Lotan Y., Shariat S.F. (2016). Current Status of Urinary Biomarkers for Detection and Surveillance of Bladder Cancer. Urol. Clin. North Am..

[23] Gontero P., Montanari E., Roupret M., Longo F., Stockley J., Kennedy A., Rodriguez O., McCracken S.R., Dudderidge T., Sieverink C. (2020). Comparison of the performances of the ADXBLADDER test and urinary cytology in the follow-up of non-muscle-invasive bladder cancer: A blinded prospective multicentric study. BJU Int..

[24] Liu Y.-L., Wang X.-L., Yang X.-H., Wu X.-H., He G.-X., Xie L.-M., Cao X.-J., Guo X.-G. (2021). Pooled analysis of Xpert Bladder Cancer based on the 5 mRNAs for rapid diagnosis of bladder carcinoma. World J. Surg. Oncol..

[25] Koya M., Osborne S., Chemasle C., Porten S., Schuckman A., Kennedy-Smith A. (2020). An evaluation of the real world use and clinical utility of the Cxbladder Monitor assay in the follow-up of patients previously treated for bladder cancer. BMC Urol..

[26] Mancini M., Righetto M., Zumerle S., Montopoli M., Zattoni F. (2020). The Bladder EpiCheck Test as a Non-Invasive Tool Based on the Identification of DNA Methylation in Bladder Cancer Cells in the Urine: A Review of Published Evidence. Int. J. Mol. Sci..

[27] Wolfs J.R.E., Hermans T.J.N., Koldewijn E.L., van de Kerkhof D. (2021). Novel urinary biomarkers ADXBLADDER and bladder EpiCheck for diagnostics of bladder cancer: A review. Urol. Oncol. Semin. Orig. Investig..

[28] Leiblich A. (2017). Recent Developments in the Search for Urinary Biomarkers in Bladder Cancer. Curr. Urol. Rep..

[29] Van Valenberg F.J.P., Hiar A.M., Wallace E., Bridge J.A., Mayne D.J., Beqaj S., Sexton W.J., Lotan Y., Weizer A.Z., Jansz G.K. (2019). Prospective Validation of an mRNA-based Urine Test for Surveillance of Patients with Bladder Cancer. Eur. Urol..

[30] Bartel D.P. (2009). MicroRNAs: Target Recognition and Regulatory Functions. Cell.

[31] Martin D., Jansson A.H.L. (2012). MicroRNA and Cancer. Mol. Oncol..

[32] Hanke M., Hoefig K., Merz H., Feller A.C., Kausch I., Jocham D., Warnecke J.M., Sczakiel G. (2010). A robust methodology to study urine microRNA as tumor marker: microRNA-126 and microRNA-182 are related to urinary bladder cancer. Urol. Oncol. Semin. Orig. Investig..

[33] Gottardo F., Liu C.G., Ferracin M., Calin G.A., Fassan M., Bassi P., Sevignani C., Byrne D., Negrini M., Pagano F. (2007). Micro-RNA profiling in kidney and bladder cancers. Urol. Oncol. Semin. Orig. Investig..

[34] Yates D.R., Rehman I., Abbod M.F., Meuth M., Cross S.S., Linkens D.A., Hamdy F.C., Catto J.W.F. (2007). Promoter Hypermethylation Identifies Progression Risk in Bladder Cancer. Clin. Cancer Res..

[35] Catto J.W.F., Abbod M.F., Wild P.J., Linkens D.A., Pilarsky C., Rehman I., Rosario D.J., Denzinger S., Burger M., Stoehr R. (2010). The Application of Artificial Intelligence to Microarray Data: Identification of a Novel Gene Signature to Identify Bladder Cancer Progression. Eur. Urol..

[36] Babjuk M., Burger M., Capoun O., Cohen D., Compérat E.M., Dominguez Escrig J.L., Gontero P., Liedberg F., Masson-Lecomte A., Mostafid A.H. (2022). European Association of Urology Guidelines on Non-muscle-invasive Bladder Cancer (Ta, T1, and Carcinoma in Situ). Eur. Urol..

[37] Laukhtina E., Shim S.R., Mori K., D‘andrea D., Soria F., Rajwa P., Mostafaei H., Compérat E., Cimadamore A., Moschini M. (2021). Diagnostic Accuracy of Novel Urinary Biomarker Tests in Non–muscle-invasive Bladder Cancer: A Systematic Review and Network Meta-analysis. Eur. Urol. Oncol..

[38] Van der Aa M.N., Steyerberg E.W., Bangma C., van Rhijn B.W., Zwarthoff E.C., van der Kwast T.H. (2010). Cystoscopy Revisited as the Gold Standard for Detecting Bladder Cancer Recurrence: Diagnostic Review Bias in the Randomized, Prospective CEFUB Trial. J. Urol..

[39] Sylvester R.J., van der Meijden A.P., Oosterlinck W., Witjes J.A., Bouffioux C., Denis L., Newling D.W., Kurth K. (2006). Predicting Recurrence and Progression in Individual Patients with Stage Ta T1 Bladder Cancer Using EORTC Risk Tables: A Combined Analysis of 2596 Patients from Seven EORTC Trials. Eur. Urol..

[40] Babjuk M., Burger M., Compérat E.M., Gontero P., Mostafid A.H., Palou J., van Rhijn B.W.G., Roupret M., Shariat S.F., Sylvester R. (2019). European Association of Urology Guidelines on Non-muscle-invasive Bladder Cancer (TaT1 and Carcinoma In Situ)—2019 Update. Eur. Urol..

[41] Mowatt G., Zhu S., Kilonzo M., Boachie C., Fraser C., Griffiths T., N’Dow J., Nabi G., Cook J., Vale L. (2010). Systematic review of the clinical effectiveness and cost-effectiveness of photodynamic diagnosis and urine biomarkers (FISH, ImmunoCyt, NMP22) and cytology for the detection and follow-up of bladder cancer. Health Technol. Assess..

[42] Compérat E., Gontero P., Liedberg F., Masson-Lecomte A., Mostafid A.H., Palou J., Van Rhijn B.W.G., Rouprêt M., Shariat S.F., Sylvester R. (2021). Non-Muscle-Invasive Bladder Cancer (TaT1 and CIS) EAU Guidelines On.

[43] Niwa N., Matsumoto K., Hayakawa N., Ito Y., Maeda T., Akatsuka S., Masuda T., Nakamura S., Tanaka N. (2015). Comparison of outcomes between ultrasonography and cystoscopy in the surveillance of patients with initially diagnosed TaG1-2 bladder cancers: A matched-pair analysis. Urol. Oncol. Semin. Orig. Investig..

[44] Roupret M., Gontero P., McCracken S.R.C., Dudderidge T., Stockley J., Kennedy A., Rodriguez O., Sieverink C., Vanié F., Allasia M. (2020). Diagnostic Accuracy of MCM5 for the Detection of Recurrence in Nonmuscle Invasive Bladder Cancer Followup: A Blinded, Prospective Cohort, Multicenter European Study. J. Urol..

[45] Pichler R., Fritz J., Tulchiner G., Klinglmair G., Soleiman A., Horninger W., Klocker H., Heidegger I. (2018). Increased accuracy of a novel mRNA-based urine test for bladder cancer surveillance. BJU Int..

[46] Witjes J.A., Morote J., Cornel E.B., Gakis G., van Valenberg F.J.P., Lozano F., Sternberg I.A., Willemsen E., Hegemann M.L., Paitan Y. (2018). Performance of the Bladder EpiCheck™ Methylation Test for Patients Under Surveillance for Non–muscle-invasive Bladder Cancer: Results of a Multicenter, Prospective, Blinded Clinical Trial. Eur. Urol. Oncol..

[47] López-Beltrán A., Cheng L., Gevaert T., Blanca A., Cimadamore A., Santoni M., Massari F., Scarpelli M., Raspollini M.R., Montironi R. (2020). Current and emerging bladder cancer biomarkers with an emphasis on urine biomarkers. Expert Rev. Mol. Diagn..

[48] Catto J.W., Alcaraz A., Bjartell A.S., White R.D.V., Evans C.P., Fussel S., Hamdy F.C., Kallioniemi O., Mengual L., Schlomm T. (2011). MicroRNA in Prostate, Bladder, and Kidney Cancer: A Systematic Review. Eur. Urol..

[49] Weber J.A., Baxter D.H., Zhang S., Huang D.Y., Huang K.H., Lee M.J., Galas D.J., Wang K. (2010). The MicroRNA Spectrum in 12 Body Fluids. Clin. Chem..

[50] Wallace E., Higuchi R., Satya M., McCann L., Sin M.L., Bridge J.A., Wei H., Zhang J., Wong E., Hiar A. (2018). Development of a 90-Minute Integrated Noninvasive Urinary Assay for Bladder Cancer Detection. J. Urol..

[51] D’elia C., Folchini D.M., Mian C., Hanspeter E., Schwienbacher C., Spedicato G.A., Pycha S., Vjaters E., Degener S., Kafka M. (2021). Diagnostic value of Xpert^®^ Bladder Cancer Monitor in the follow-up of patients affected by non-muscle invasive bladder cancer: An update. Ther. Adv. Urol..

[52] D´elia C., Pycha A., Folchini D.M., Mian C., Hanspeter E., Schwienbacher C., Vjaters E., Pycha A., Trenti E. (2019). Diagnostic predictive value of Xpert Bladder Cancer Monitor in the follow-up of patients affected by non-muscle invasive bladder cancer. J. Clin. Pathol..

[53] Cancel-Tassin G., Roupret M., Pinar U., Gaffory C., Vanie F., Ondet V., Compérat E., Cussenot O. (2021). Assessment of Xpert Bladder Cancer Monitor test performance for the detection of recurrence during non-muscle invasive bladder cancer follow-up. World J. Urol..

[54] Hurle R., Casale P., Saita A., Colombo P., Elefante G.M., Lughezzani G., Fasulo V., Paciotti M., Domanico L., Bevilacqua G. (2020). Clinical performance of Xpert Bladder Cancer (BC) Monitor, a mRNA-based urine test, in active surveillance (AS) patients with recurrent non-muscle-invasive bladder cancer (NMIBC): Results from the Bladder Cancer Italian Active Surveillance (BIAS) project. World J. Urol..

[55] Pichler R., Fritz J., Tulchiner G., Klinglmair G., Soleiman A., Horninger W., Klocker H., Heidegger I., Wallace E., Higuchi R.G. (2021). Prospective Validation of an MRNA-Based Urine Test for Surveillance of Patients with Bladder Cancer. Eur. Urol..

[56] Lotan Y., Roehrborn C.G. (2003). Sensitivity and specificity of commonly available bladder tumor markers versus cytology: Results of a comprehensive literature review and meta-analyses. Urology.

[57] Benderska-Söder N., Hovanec J., Pesch B., Goebell P.J., Roghmann F., Noldus J., Rabinovich J., Wichert K., Gleichenhagen J., Käfferlein H.U. (2020). Toward noninvasive follow-up of low-risk bladder cancer—Rationale and concept of the UroFollow trial. Urol. Oncol. Semin. Orig. Investig..

[58] Kamat A.M., Karakiewicz P.I., Xylinas E., Hegarty P.K., Hegarty N., Jenkins L.C., Droller M., van Rhijn B.W., Shariat S.F., Schmitz-Dräger B.J. (2012). ICUD-EAU International Consultation on Bladder Cancer 2012: Screening, Diagnosis, and Molecular Markers. Eur. Urol..

[59] Lotan Y., Roehrborn C.G. (2002). Cost-effectiveness of a modified care protocol substituting bladder tumor markers for cystoscopy for the follow up of patients with transitional cell carcinoma of the bladder: A decision analytical approach. J. Urol..

[60] Nam R.K., Redelmeier D.A., Spiess P.E., Sampson H.A., Fradet Y., Jewett M.A.S. (2000). Comparison of molecular and conventional strategies for follow up of superficial bladder cancer using decision analysis. J. Urol..

[61] Van der Aa M.N., Steyerberg E.W., Sen E.F., Zwarthoff E.C., Kirkels W.J., van der Kwast T.H., Essink-Bot M.-L. (2008). Patients’ perceived burden of cystoscopic and urinary surveillance of bladder cancer: A randomized comparison. BJU Int..

[62] Koo K., Zubkoff L., Sirovich B.E., Goodney P.P., Robertson D.J., Seigne J.D., Schroeck F.R. (2017). The Burden of Cystoscopic Bladder Cancer Surveillance: Anxiety, Discomfort, and Patient Preferences for Decision Making. Urology.

[63] Burger M., Grossman H.B., Droller M., Schmidbauer J., Hermann G., Drăgoescu O., Ray E., Fradet Y., Karl A., Burgués J.P. (2013). Photodynamic Diagnosis of Non–muscle-invasive Bladder Cancer with Hexaminolevulinate Cystoscopy: A Meta-analysis of Detection and Recurrence Based on Raw Data. Eur. Urol..

[64] Van Osch F.H.M., Nekeman D., Aaronson N.K., Billingham L.J., James N.D., Cheng K.K., Bryan R.T., Zeegers M.P. (2019). Patients choose certainty over burden in bladder cancer surveillance. World J. Urol..

[65] Tan W.S., Teo C.H., Chan D., Heinrich M., Feber A., Sarpong R., Allan J., Williams N., Brew-Graves C., Ng C.J. (2019). Mixed-methods approach to exploring patients’ perspectives on the acceptability of a urinary biomarker test in replacing cystoscopy for bladder cancer surveillance. BJU Int..

[66] Sylvester R.J., Oosterlinck W., Holmang S., Sydes M.R., Birtle A., Gudjonsson S., De Nunzio C., Okamura K., Kaasinen E., Solsona E. (2016). Systematic Review and Individual Patient Data Meta-analysis of Randomized Trials Comparing a Single Immediate Instillation of Chemotherapy After Transurethral Resection with Transurethral Resection Alone in Patients with Stage pTa–pT1 Urothelial Carcinoma of the Bladder: Which Patients Benefit from the Instillation?. Eur. Urol..

[67] Palou J., Brausi M., Catto J.W. (2020). Management of Patients with Normal Cystoscopy but Positive Cytology or Urine Markers. Eur. Urol. Oncol..

[68] Cowan B., Klein E., Jansz K., Westenfelder K., Bradford T., Peterson C., Scherr D., Karsh L.I., Egerdie R.B., Witjes J.A. (2021). Longitudinal follow-up and performance validation of an mRNA-based urine test (Xpert ^®^ Bladder Cancer Monitor) for surveillance in patients with non-muscle-invasive bladder cancer. BJU Int..

[69] Todenhöfer T., Hennenlotter J., Guttenberg P., Mohrhardt S., Kuehs U., Esser M., Aufderklamm S., Bier S., Harland N., Rausch S. (2015). Prognostic relevance of positive urine markers in patients with negative cystoscopy during surveillance of bladder cancer. BMC Cancer.

[70] Gopalakrishna A., Fantony J.J., Longo T.A., Owusu R., Foo W.-C., Dash R., Denton B.T., Inman B.A. (2017). Anticipatory Positive Urine Tests for Bladder Cancer. Ann. Surg. Oncol..

